# Activation of IGF-1/GLP-1 Signalling via 4-Hydroxyisoleucine Prevents Motor Neuron Impairments in Experimental ALS-Rats Exposed to Methylmercury-Induced Neurotoxicity

**DOI:** 10.3390/molecules27123878

**Published:** 2022-06-16

**Authors:** Ambika Shandilya, Sidharth Mehan, Sumit Kumar, Pranshul Sethi, Acharan S. Narula, Abdulrahman Alshammari, Metab Alharbi, Abdullah F. Alasmari

**Affiliations:** 1 Division of Neuroscience, Department of Pharmacology, ISF College of Pharmacy, Moga 142001, Punjab, India; ambika07082015@gmail.com (A.S.); sumitsrivastav223@gmail.com (S.K.); pranshulsethiptl@gmail.com (P.S.); 2 Narula Research, LLC, 107 Boulder Bluff, Chapel Hill, NC 27516, USA; acharannarula@icloud.com; 3 Department of Pharmacology and Toxicology, College of Pharmacy, King Saud University, Post Box 2455, Riyadh 11451, Saudi Arabia; abdalshammari@ksu.edu.sa (A.A.); mesalharbi@ksu.edu.sa (M.A.); afalasmari@ksu.edu.sa (A.F.A.)

**Keywords:** amyotrophic lateral sclerosis, GLP-1, IGF-1, methylmercury, motor neuron death, 4-hydroxyisoleucine, demyelination

## Abstract

Amyotrophic lateral sclerosis (ALS) is a severe adult motor neuron disease that causes progressive neuromuscular atrophy, muscle wasting, weakness, and depressive-like symptoms. Our previous research suggests that mercury levels are directly associated with ALS progression. MeHg+-induced ALS is characterised by oligodendrocyte destruction, myelin basic protein (MBP) depletion, and white matter degeneration, leading to demyelination and motor neuron death. The selection of MeHg+ as a potential neurotoxicant is based on our evidence that it has been connected to the development of ALS-like characteristics. It causes glutamate-mediated excitotoxicity, calcium-dependent neurotoxicity, and an ALS-like phenotype. Dysregulation of IGF-1/GLP-1 signalling has been associated with ALS progression. The bioactive amino acid 4-hydroxyisoleucine (HI) from Trigonella foenum graecum acts as an insulin mimic in rodents and increases insulin sensitivity. This study examined the neuroprotective effects of 4-HI on MeHg+-treated adult Wistar rats with ALS-like symptoms, emphasising brain IGF1/GLP-1 activation. Furthermore, we investigated the effect of 4-HI on MBP levels in rat brain homogenate, cerebrospinal fluid (CSF), blood plasma, and cell death indicators such as caspase-3, Bax, and Bcl-2. Rats were assessed for muscular strength, locomotor deficits, depressed behaviour, and spatial learning in the Morris water maze (MWM) to measure neurobehavioral abnormalities. Doses of 4-HI were given orally for 42 days in the MeHg+ rat model at 50 mg/kg or 100 mg/kg to ameliorate ALS-like neurological dysfunctions. Additionally, neurotransmitters and oxidative stress markers were examined in rat brain homogenates. Our findings suggest that 4-HI has neuroprotective benefits in reducing MeHg+-induced behavioural, neurochemical, and histopathological abnormalities in ALS-like rats exposed to methylmercury.

## 1. Introduction

Amyotrophic lateral sclerosis (ALS) is an idiopathic, chronic, progressive motor neuron disease (MND) characterised by severe neurological dysfunction [1]. The several neuropathogenic processes that lead to ALS include neuronal apoptosis [2], oligodendrocyte destruction, chronic inflammatory reactions, and demyelination [3].

MeHg+ is a potent neurotoxin that causes neurocognitive deficits, mimicking an experimental animal model of ALS in adult Wistar rats [1,4]. Several studies have found that MeHg+ causes a variety of neuro-complications, including increased intracellular ROS levels, while reducing the expression of the anti-apoptotic Bcl-2 protein in PC12 cells [5]. MeHg+ has also been found to cause hippocampal damage, learning and memory deficits [6], neurobehavioural impairment [7], neuroinflammation [8], and motor cortex degeneration [9].

IGF-1/GLP-1 are trophic factors that modulate neuronal homeostasis and neuroplasticity [10]. They are required for neuronal proliferation, growth, and differentiation [11,12,13]. IGF-1/GLP-1 signal transduction is also involved in neural metabolism, oligodendrogenesis [14], myelin sheath formation [15], synaptic transmission, axonal development, and regeneration [16]. Downregulation of their signalling results in neuroexcitation and neuroinflammation [17] as well as various neuropathological features associated with ALS progression [18].

Numerous studies in preclinical and clinical models targeting insulin/IGF-1 and GLP-1 signalling suggest that cohesive activation of both targets can positively reverse insulin deregulation and elicit neuroprotective effects [19]. Increasingly; research has shown that insulin administration and IGF-1/GLP-1 activators play a critical role in reversing numerous pathological dysfunctions in ALS.

IGF-1, recombinant human IGF-1 (rh-IGF-1), and insulin bind to the IGF-1R and prevent neuronal death [20], misfolded protein aggregation in the neuronal cytoplasm [21], astrogliosis [22], and neuroinflammation [23]. GLP-1 and its analogues reduce neurological dysfunctions by promoting neurogenesis through cAMP production [24], enhancing neurotransmission [25], and promoting dendritic morphogenesis [26] as well as synaptic plasticity [27]. Furthermore, these analogues prevent inappropriate microglia-mediated cytokine production, apoptosis [28,29], oxidative stress [30], and axonal transport abnormalities [31].

The α-amino acid 4-hydroxyisoleucine (4-HI) is naturally occurring, non-proteinogenic, and has a branched-chain. It is derived from the Leguminosae plant Trigonella foenum graecum (fenugreek seeds) [32]. It has a high therapeutic potential for treating insulin dysregulation [33]. Reportedly, 4-HI has therapeutic benefits against insulin resistance [34], insufficient glucose absorption [35], lipotoxicity [36], obesity [37], inflammation [38,39], and liver dysfunctions [40]. A 50 mg/kg dose of 4-HI increased insulin secretion and had anti-diabetic effects in a rat model of non-insulin-dependent diabetes [41]. In addition, 4-HI has demonstrated effectiveness as a treatment for post-brain haemorrhage and neurochemical alterations associated with neurological impairment [42]. Researchers have investigated 4-HI’s antidepressant-like effects in mice models of depression (10, 30, or 100 mg/kg) [40,43,44]. Previous research found that 4-HIenhanced serotonin turnover in the brain [45]. Furthermore, 4-HI (50, 100, and 200 mg/kg) has been shown to provide long-term protection against thermal hyperalgesia and motor function impairments [46].

Recently, Mehan and co-workers successfully explored the neuroprotective potential of 4-HI in intracerebral haemorrhage [47]. Previous studies have demonstrated that 4-HI dose-dependently promotes GLP-1-dependent cAMP production and GLP-1R endocytosis [48]. Furthermore, 4-Hydroxyisoleucine (4-HI) enhances insulin receptor-β and insulin receptor substrate-1 tyrosine phosphorylation. Additionally, 4-HI activates the AMP-activated protein kinase and PI3-kinase pathways, which are required for nerve growth factor (NGF) and brain-derived neurotrophic factor (BDNF) neuroprotective activities [49].

There is no particular treatment for curing ALS in the present scenario, nor are there associated biomarkers. Moreover, based on the above findings, we hypothesised that 4-HI could enhance IGF-1/GLP-1 target signalling mechanisms and attenuate neurodegenerative changes in MeHg+-induced experimental rats. Further, 4-HI could be combined with other standard pharmacological therapies to improve the lives of people with demyelinating diseases like ALS. Therefore, in the current research, we investigated and evaluated 4-HI’s neuroprotective profile in rat brain homogenate, blood plasma, and CSF samples to see if these biological samples may be valuable and repeatable diagnostic biomarkers during the early stages of neurological degeneration.

## 2. Material and Methods

### 2.1. Experimental Animals

The Central Animal House, ISF College of Pharmacy, Moga, Punjab, provided 36 adult Wistar rats (250–300 g, aged six months, total animals = 36, total experiment groups = 06, each group comprising six rats of either sex). The animals were kept in polyacrylic cages (38 cm×32 cm×16 cm; 3–4 rats per cage) with a wire mesh top and soft paddy husk bedding. They were acclimatized to standard husbandry conditions of a 12 h light/dark constant cycle with a normal chow diet as dried pellets and filtered water ad libitum, maintained at a controlled temperature of 23 ± 2 and relative humidity 65–70%. The experimental protocol was approved by the Institutional Animal Ethics Committee (IAEC) with registration no. 816/PO/ReBiBt/S/04/CPCSEA and protocol no. ISFCP/IAEC/CPCSEA/Meeting No.27/2020/Protocol No.455 in compliance with the guidelines of the Government of India. Animals were adapted for a week to standard laboratory conditions before the commencement of the experiments.

### 2.2. Chemicals and Drugs

MeHg+ was purchased from Sigma-Aldrich (St. Louis, MO, USA). The test drug, 4-HI (amorphous, hydrophilic powder), was acquired as an ex-gratia sample from BAPEX Pharmaceuticals, India. All of the other compounds employed in the study were of analytical grade. An aqueous solution of 4-HI with 2% ethanol was administered in 10 mL/kg volume via oral gavage [42,45].

### 2.3. Experimental Animal Model of Methylmercury-Induced ALS-Like Rats

For the last 13 years, Mehan and co-workers in the Division of Neuroscience at ISF College of Pharmacy, Moga, have been continuously developing drug discovery screening procedures and finding diagnostic biomarkers involved in neurodegenerative and neuropsychiatric problems. The MeHg+-induced experimental model of ALS in rats was established according to the method validated by Mehan and co-workers [4]. Experimental rats were administered MeHg+ via oral gavage at 5 mg/kg for three consecutive weeks. Pre-clinically, MeHg+ causes neurological impairment similar toan experimental animal model of ALS and is considered a valid model for studying pathophysiological changes similar to ALS [1]. Thus, in this study, MeHg+ was used as a neurotoxin to observe behavioural impairments, neurochemical imbalances, and gross morphological alterations in ALS-like rats.

### 2.4. Protocol Schedule of Animal Experimentation

**Figure 1:**
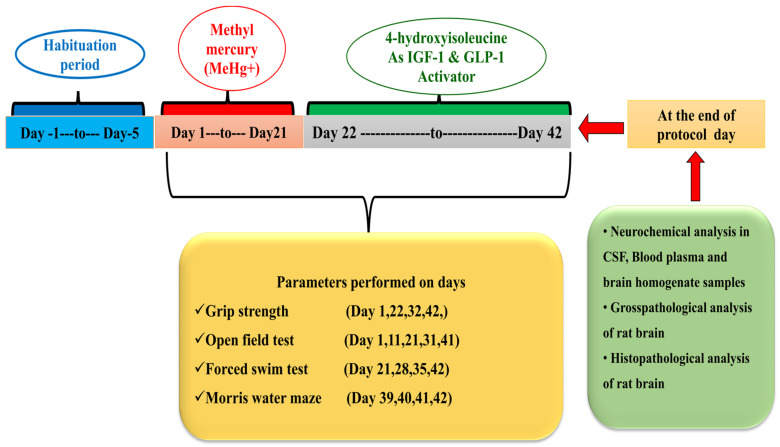
Experimental protocol schedule.

The total duration of the experimental protocol schedule was 42 days. From day 1 to 21, MeHg+ was regularly administered via oral gavage at the dose of 5 mg/kg. The test drug, 4-HI, was administered through an oral cannula at the dose of 50 mg/kg, and 100 mg/kg from day 22 until the 42nd day of the study duration. Animals were randomly assigned to six groups (*n* = 6 animals per group). Group 1: Normal control, Group 2: Vehicle control, Group 3: (4-HI100 mg/kg, *perse, p.o.*), Group 4: (MeHg5 mg/kg, *p.o.*), Group 5: (MeHg5 mg/kg, *p.o.* + 4-HI50 mg/kg, *p.o.*), and Group 6: (MeHg5 mg/kg, *p.o.* + 4-HI100 mg/kg, *p.o.*). The vehicle control group was employed in the study to determine whether the vehicle (water with 2% ethanol) alone causes any effects compared with untreated normal control. Various neurobehavioral assessments like grip strength test, open field test, forced swim test, and Morris water maze task were carried out between days 1 and 42 of the study duration to evaluate the alteration in behaviours like learning, memory, and emotion brain function in different experimental groups. At the end of the protocol schedule, i.e., day 43rd, animals were deeply anaesthetised with sodium pentobarbital (270 mg/mL, i.p.), and 2.5 mL of blood was collected from the rats through a retro-bulbar puncture [50], and 100 µL of CSF was collected from the cistern Magna and stored at −80 °C for future use. Animals were then sacrificed by decapitation, and their whole brains were carefully removed from the skull for various neurochemical estimations and gross morphological analysis. Fresh whole brains isolated from the rats were weighed and further homogenised in phosphate buffer saline (PBS). All the biochemical estimations were performed in brain homogenate, CSF, and blood plasma samples. Figure 1 shows a schematic representation of the protocol paradigm and time course of experimentation.

### 2.5. Parameters Evaluated

#### 2.5.1. Assessment of Weight Variations

##### Assessment of Body Weight

Body weight alteration was monitored on days 1, 7, 14, 21, 28, 35 and 42nd of the experiment schedule [51].

##### Assessment of Relative Brain–Body Weight Ratio

On the 42nd day of the experimental schedule, the brain weights of all rats were recorded after their sacrification. A brain-to-final body weight ratio was calculated to examine the alterations in absolute brain mass of MeHg treated ALS-like rats with respect to their body weight. This assessment can detect the chronic, neurotoxic effects of MeHg exposure on the brain and hence can potentially establish a link between MeHg exposure and loss of brain–body weight. The relative brain–body weight ratio was calculated using the formula (brain weight/body weight) ×100 [52].

### 2.6. BehaviourParameters

#### 2.6.1. Grip Strength Test (GST)

In different treatment groups, a grip strength test was used to evaluate forelimb and hind limb strength. The test was performed on days 1, 22, 33 and 42 of the protocol. The rats were allowed to grasp the metal bar/grid with their forepaws and were then pulled backwards by the tail in the horizontal plane. The force applied to the grid before the rat lost grip was recorded as the maximal peak force. This force was measured in kgf (Chatillon, NJ, USA) [53].

#### 2.6.2. Open Field Test (OFT) Assessment

Locomotor activity and anxiety-like behaviour in the rats were assessed with the open-field task. OFT assessment is a standard measure of rodents’ exploratory behaviour and general locomotor activity. Experimental animals were tested for their locomotor activity and number of rearings on the 1st, 11th, 21st, 31st, and 41sttreatment days. Each animal was placed at the centre of the enclosure (70 cm long× 70 cm wide× 60 cm high), and the number of boxes/segments crossed by the animal with their four paws in five minutes was recorded as a measure of their locomotor activity. The number of rearing outcomes indicated by the animal throughout the five-minute sessions was evaluated as an indicator of emotionality (degree of anxiety) [1].

#### 2.6.3. Forced Swim Test (FST)

FST is a behavioural despair test used to evaluate depressive-like behaviour in animals. It measures an animal’s susceptibility to the acute stress of water by assessing its response to the threat of drowning. Each animal was tested for a forced swim on days 21, 28, 35 and 42 of the study by placing them individually in a transparent, plastic cylindrical tank filled up to 15 cm with tap water (at 23–25°C temperature; height: 40 cm; diameter: 18 cm). The immobility time and swimming time (movement usually horizontal throughout the swim chamber) were manually scored in each trial for five minutes. The rats were scored for immobility duration when they ceased struggling and remained floating motionless in the upright position by making only small movements to keep their head above water. Immediately following the test, the rats were wrapped in a dry towel and placed under a heating lamp to dry [54].

#### 2.6.4. Morris Water Maze (MWM) Task

The MWM test is a robust and reliable test strongly correlated with hippocampal synaptic plasticity and cognitive dysfunctions. This task was performed to evaluate spatial learning and memory in rats [47]. The working memory of the rats was analysed by assessing their escape latency time (ELT) on the protocol schedule’s 39th, 40th and 41st days. Rats were allowed to navigate freely around the perimeter of an open swimming arena to locate a submerged escape platform. Twenty-four hours after the acquisition phase, the time taken by the rats to find a platform and escape the maze was recorded as ELT (long-term memory). 

On the 42nd day, a probe/memory test was conducted by removing the platform. The rats were allowed to swim freely in the pool of water to find the platform (previously present) within 120 s. The time spent by the animal in the target quadrant (TSTQ) zone to locate the platform was recorded by a video system. The TSTQ indicated the degree of relative memory consolidation (reference memory) in rats following spatial learning [55].

### 2.7. Neurochemical Parameters

#### 2.7.1. Collection and Preparation of Biological Samples

On day 43 of the experiment, 2.5 mL of blood was collected from anaesthetized rats through retro-bulbar puncture from the orbital venous plexus by inserting a capillary tube medially into the rat eye. Blood from the plexus was collected into a sterile Eppendorf tube containing EDTA via the capillary action through gentle rotation and retraction [56]. The freshly drawn blood samples were centrifuged at 10,000× *g* for 15 min to separate the plasma, and the supernatant was carefully stored in deep freeze (at −80 °C) for further use.

Following blood collection, rats were deeply anaesthetized with sodium pentobarbital (270 mg/mL, i.p.) and subjected to a caudal incision, translucent duramater was exposed, and a 30-gauge needle was gently placed at a 30° angle into the cisterna magna [57]. Approximately 100 µL CSF was carefully ejected into a 0.5 mL sterile Eppendorf tube using the suction pressure of a 1 mL tuberculin syringe attached to a needle. The collected sample was frozen at 80 °C until analysis with ELISA [58].

Immediately after CSF collection, rats were sacrificed by decapitation; whole brains were isolated from the skull with the utmost care, freshly weighed, and washed with ice-cold, isotonic saline solution, and then homogenized with 0.1 M (*w*/*v*) of chilled PBS (pH = 7.4). The rat brain homogenate was centrifuged at 10,000× *g* for 15 min, the supernatant was separated, and the aliquots were preserved. The samples were deep-frozen at −80 °C to be used as and when required for various biochemical estimations.

#### 2.7.2. Assessment of Cellular and Molecular Markers

The concentration of IGF-1 was determined in rat brain homogenate [59] and CSF [60] using ELISA commercial kits (E-EL-R0010/IGF-1; E-EL-R3007/GLP-1; E-EL-R0642/MBP; Elabsciences, Wuhan, Hubei, China) as per the manufacturer’s instructions. Similarly, GLP-1 levels were assessed in brain homogenate [61], and CSF samples were obtained from rats. Furthermore, the level of myelin basic protein (MBP) was measured in rat brain homogenate [4] and CSF samples [62]. The levels of IGF-1 and GLP-1 proteins in brain homogenate and CSF were represented as pg/mg protein and ng/mL protein, respectively. For MBP, the values were expressed as µg/mg protein in brain homogenate and ng/L protein in CSF.

#### 2.7.3. Assessment of Apoptotic Markers

The level of pro-apoptotic markers like caspase-3, Bax, and anti-apoptotic Bcl-2 was assessed using ELISA assay kits (E-EL-R0160/Caspase-3; E-EL-R0098/Bax/Bcl2Elabsciences, Wuhan, China). As per the given standard procedures, quantification tests for caspase-3 were performed in rat brain homogenate [63] and blood plasma [64]. Similarly, Bax level was measured in brain homogenate [65] and blood plasma samples [66]. Moreover, the concentration of Bcl-2 protein was also evaluated in rat brain homogenate [67] and blood plasma [68] obtained from rats. The caspase-3 level was measured in brain homogenate as nM/mg protein and blood plasma as ng/mL [47]. The level of Bax/Bcl-2 in brain homogenate and blood plasma were represented in ng/mg and ng/mL, respectively.

#### 2.7.4. Assessment of Neurotransmitter Levels

Glutamate is the major excitatory amino acid neurotransmitter in the CNS, playing a crucial role in brain development, learning, and memory. At first, the derivatisation of ortho-phthalaldehyde/β-mercaptoethanol was performed, followed by quantifying GABA and glutamate levels. The method of Alam and co-workers was followed for this quantitative analysis of the brain tissue samples. The amount of GABA/glutamate in the brain homogenate supernatant was expressed as ng/mg protein [69].

A diagnostic kit (KRISHGEN Diagnostics, Mumbai, India) was used to measure acetylcholine (Ach) levels. All the reagents and samples were freshly prepared as described in the kit. The optical density of the reaction mixture was determined at 540 nm with the help of a microtiter plate. The level of Ach present in the brain homogenate was expressed as ng/mg protein [70].

The serotonin (5-HT) level was estimated with the help of HPLC using a C18 reversed-phase column and an electrochemical detector (ECD) at +0.75 V, with sensitivity ranging from 5 to 50 nA. The mobile phase consisted of sodium citrate buffer (pH = 4.5) and acetonitrile in the ratio 87:13, *v*/*v*. The concentration of serotonin was estimated using a standard with a concentration of 10–100 mg/mL from the standard curve. The level of the 5-HT present in the rat brain homogenate was expressed as ng/mg protein [65].

#### 2.7.5. Assessment of Inflammatory Cytokine Levels

The level of TNF-α was quantified in brain homogenate [71] and blood plasma [72] by using rat TNF-α immunoassay kits (E-EL-R0019/TNF-α; E-EL-R0012/IL-1β; Elabsciences, Wuhan, China). Similarly, using ELISA commercial kits, the concentration of IL-1β was also determined in rat brain homogenate [73] and blood plasma samples [74]. These inflammatory cytokine levels in rat brain homogenate and blood plasma were expressed as pg/mg protein and pg/mL protein, respectively.

#### 2.7.6. Assessment of Oxidative Stress Markers

To measure the level of LDH in the rat brain homogenate, a diagnostic kit (Transasia Bio-Medicals Ltd., Mumbai, India) was used. The amount of LDH was quantified as Units/L [75,76].

The amount of MDA in the homogenate was determined using the spectrometric method at 532 nm after reaction with the thiobarbituric acid (TBA) reagent. The TBARS concentration was calculated using TEP as a standard. The MDA concentration was expressed as nM/mg protein [77].

The nitrite (NO_2_) level was determined by a colourimetric assay using a Greiss reagent, and the absorbance was determined at 540 nm with a UV spectrometer. The nitrite concentration in the supernatant was determined via a sodium nitrite standard curve and expressed as µM/mg protein [47].

The amount of antioxidant SOD was quantified based on the ability of SOD to inhibit the self-oxidation of epinephrine in alkaline conditions. Coloured adrenochrome is formed during this reaction, while superoxide anion-radicals are formed as intermediate products of this process, and the absorbance was measured with a spectrometer at 480 nm. The level of SOD in the sample was expressed as µM/mg protein [78].

The antioxidant GSH content in brain homogenate was determined based on the concept of DTNB reduction by thiol compounds (mainly GSH) to coloured 2-nitro-5-mercaptobenzoic acid. The developed yellow colour was measured immediately at 412 nm using a UV spectrometer. The concentration of GSH in the supernatant was expressed as µM/mg protein [79].

#### 2.7.7. Assessment of Acetylcholinesterase Enzyme Level

The AchE level in the brain homogenate sample was determined spectrophotometrically. The assay mixture contained brain homogenate supernatant, 0.01 M sodium phosphate buffer (pH = 8), acetylthiocholine iodide, and DTNB (Ellman’s reagent). The enzymatic activity of the supernatant was expressed as µM/mg protein [80].

#### 2.7.8. Assessment of Gross Pathology and Demyelination Volume in Rat Brain

Animals were decapitated and sacrificed on day 43 after completing the experimental schedule. Fresh, whole brains were isolated from the skull, freshly weighed, and immediately preserved in ice-cold saline for gross pathological analysis. Coronal brain sections were taken after a macroscopic examination of the whole rat brain [81]. Cerebral hemispheres were sectioned into 2 mm thick parallel coronal slices and placed on glass slides for morphological and pathological assessment. A digital camera was used for macroscopic inspection of the entire striatum [67]. The volume of the demyelinated region (mm^3^) for each brain segment was estimated from the greyish region around the striatum. The extent of white matter degeneration in each coronal segment was determined by calculating the demyelination volume (length × breadth × height) [4].

#### 2.7.9. Assessment of Histopathological Changes

Once the experimental protocol was finished, the animals were anaesthetised with sodium phenobarbital (270 mg/mL, i.p.) and sacrificed by decapitation. The cerebral cortex was delicately removed from the whole brain for histological investigation. The isolated area was thoroughly cleaned before slicing into 0.5 cubic centimetre slices. Following a further fixation in 4% paraformaldehyde in PBS PH = 7.4 overnight (8–12h) at room temperature, immersion in 70% ethanol was performed. The tissue was kept at 37°C until it was ready to be embedded in paraffin wax. The paraffin blocks were chopped into 4–5 m thickness using a rotary microtome. Hematoxylin and eosin staining was used on the sections, and morphology was analysed using a fluorescent microscope (Type 102 M;100 × Magnification). The properties and density of the normal neuronal population (Oligodendrocytes, microglial cells, and area of necrosis) in the cerebral cortex were observed blindly using a fluorescent microscope with a reticular consolidated eyepiece at a magnification of 100× [29].

### 2.8. Statistical Analysis

Data generated were analysed using two-way analysis of variance (ANOVA) followed by post-hoc Bonferroni’s test, and one-way ANOVA repeated measures followed by post-hoc Tukey’s multi comparison test to examine the differences between various treatment groups. Two-way ANOVA was used to analyse the body weight and behavioural parameters. In contrast, one-way ANOVA was used to analyse the relative brain–body weight ratio, demyelination volume, biochemical parameters, and TSTQ analysis. *p* < 0.001 was considered statistically significant. Data was found to be normal, and the sample size was calculated by checking the normality distribution via the Kolmogorov–Smirnov test. The statistical analysis was performed using GraphPad Prism software version 5.03 for Windows (GraphPad Software, San Diego, CA, USA). The statistical results are presented as the mean and standard error of mean (SEM).

## 3. Results

### 3.1. Effect of 4-Hydroxyisoleucine in the Restoration of Weight Variations after Methyl Mercury-Exposure in Rats

#### 3.1.1. Improved Body Weight after Long-Term Administration of 4-Hydroxyisoleucine

**Figure 2:**
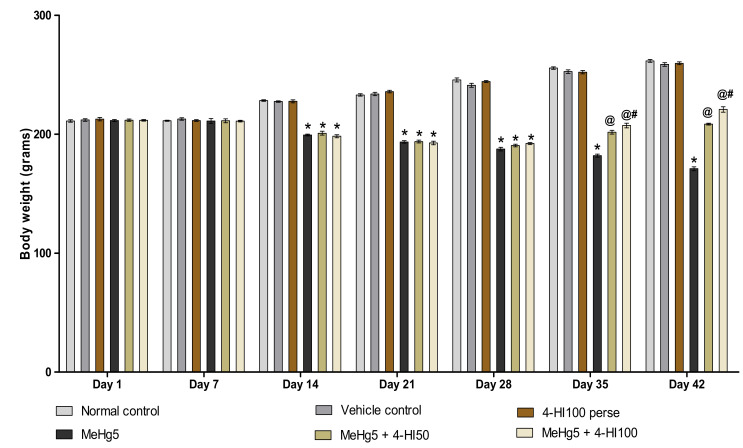
Effect of 4-hydroxyisoleucine on body weight after methylmercuryexposure in rats. Statistical analysis by two-way ANOVA (posthoc Bonferroni’s test). Values are expressed as mean ± SEM (*n* = 6 rats per group). * *p* < 0.001 v/s normal control, vehicle control and 4-HI100 *perse*; @ *p* < 0.001 v/s MeHg5; @# *p* < 0.001 v/s MeHg5 + 4-HI50.

To determine the role of 4-HI in MeHg-treated rats, alterations in body weight were assessed on days 1, 7, 14, 21, 28, 35 and 42 of the experimental protocol schedule. On days 1and 7, there was no significant difference between the groups. On days 14, 21, 28, 35 and 42, a gradual decline in bodyweight was found in rats treated with MeHg compared to normal control, vehicle control, and 4-HI100 *perse* treated groups. Continuous oral administration of 4-HI50 mg/kg and 100 mg/kg significantly and dose-dependently improved the bodyweight when compared with MeHg-treated rats on the 14th, 21st, 28th, 35th and 42nd days of the experimental schedule (two-way ANOVA: F(30,180) = 187.8, *p* < 0.001). On days 35 and 42, 4-HI100 mg/kg treated rats showed significant improvements in body weight restoration compared to the 4-HI50 mg/kg treatment rats (Figure 2).

#### 3.1.2. Improvement in Relative Brain–Body Weight Ratio after Long-Term Administration of 4-Hydroxyisoleucine

**Figure 3:**
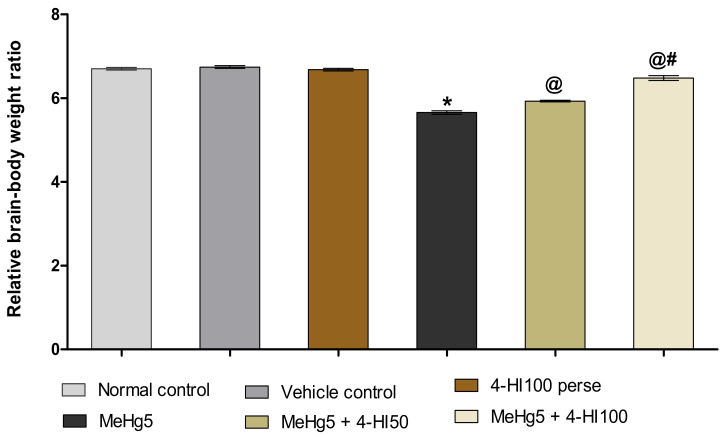
Effect of 4-hydroxyisoleucine on relative brain–body weight ratio after methyl mercury-exposure in rats. Statistical analysis by one-way ANOVA (posthoc Tukey’s test). Values are expressed as mean ±SEM (*n* = 6 rats per group). * *p* < 0.001 v/s normal control, vehicle control and 4-HI100 *perse*; @ *p* < 0.001 v/s MeHg5; @# *p* < 0.001 v/s MeHg5 + 4-HI50.

On the 42nd day of the experimental procedure, the relative brain–body ratio was analysed to investigate the neuroprotective effect of 4-HI in MeHg-treated rats. The normal and vehicle control groups showed no significant difference in the relative brain–body weight ratio compared to 4-HI *perse* treated rats. Moreover, compared to the normal control, vehicle control, and 4-HI100 *perse* groups, MeHg-induced rats demonstrated a steady decline in relative brain–body weight ratio. Long-term oral administration of 4-HI at the doses of 50 mg/kg and 100 mg/kg substantially enhanced the relative brain–body weight ratio compared to the MeHg-treated rats (one-way ANOVA: F(5,25) = 0.632, *p* < 0.001). Compared to the 4-HI 50 mg/kg treated group, 4-HI100 mg/kg demonstrated a considerable increase in relative brain–body weight ratio. These results reflect that 4-HI can potentially restore the absolute brain mass alterations with respect to body weight (Figure 3).

### 3.2. Effect of 4-Hydroxyisoleucine in the Amelioration of Neurobehavioral Abnormalities after Methyl Mercury Exposure in Rats

#### 3.2.1. Improved Grip Strength after Long-Term Administration of 4-Hydroxyisoleucine

**Figure 4:**
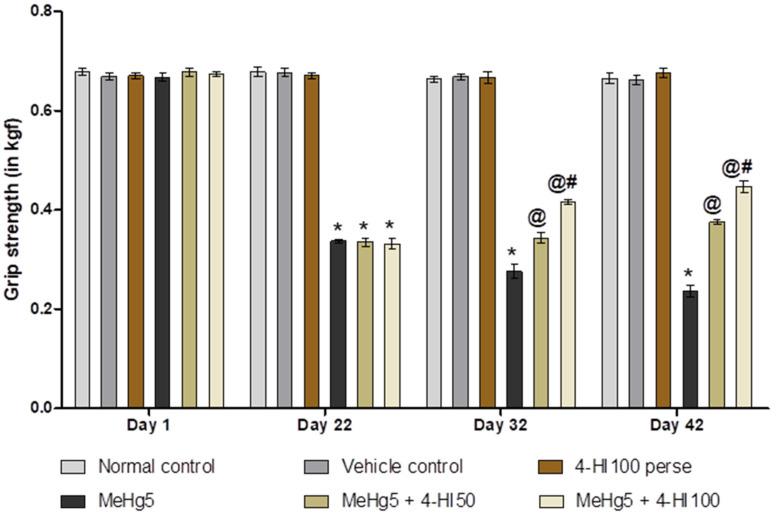
Effect of 4-hydroxyisoleucine on grip strength after methylmercury exposure in rats.Statistical analysis by two-way ANOVA (posthoc Bonferroni’s test). Values are expressed as mean ± SEM (*n* = 6 rats per group). * *p* < 0.001 v/s normal control, vehicle control and 4-HI100 perse; @ *p* < 0.001 v/s MeHg5; @# *p* < 0.001 v/s MeHg5 + 4-HI50.

Grip strength tests were performed on days 1, 22, 32 and 42 of the protocol schedule to assess the potential influence of 4-HI on muscle strength. On day 1, a non-significant difference was observed between all the treatment groups. The oral treatment with MeHg for 21 days consecutively led to a gradual decrease in grip strength force on days 22, 32 and 42 compared to normal control, vehicle control, and 4-HI100 *perse* groups. Prolonged administration of 4-HI 50 mg/kg and 100 mg/kg showed a remarkable improvement in grip strength force on days 22, 32 and 42 when compared to MeHg-treated rats (two-way ANOVA: F(15,90) = 119.6, *p* < 0.001). Among these, 4-HI100 mg/kg was more effective than 4-HI 50 mg/kg in the restoration of grip strength on days 32 and 42 (Figure 4).

#### 3.2.2. Improved Locomotion and Restored Anxiety-Like Behaviour after Long-Term Administration of 4-Hydroxyisoleucine

**Figure 5:**
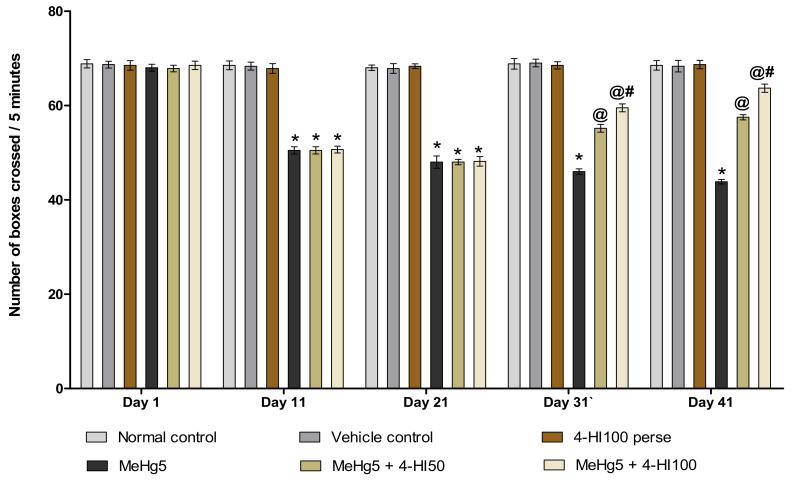
Effect of 4-hydroxyisoleucine on locomotion activity (number of boxes crossed) after methyl mercuryexposure in rats. Statistical analysis by two-way ANOVA (posthoc Bonferroni’s test). Values are expressed as mean ± SEM (*n* = 6 rats per group). * *p* < 0.001 v/s normal control, vehicle control and 4-HI100 *perse*; @ *p* < 0.001 v/s MeHg5; @# *p* < 0.001 v/s MeHg5 + 4-HI50.

**Figure 6:**
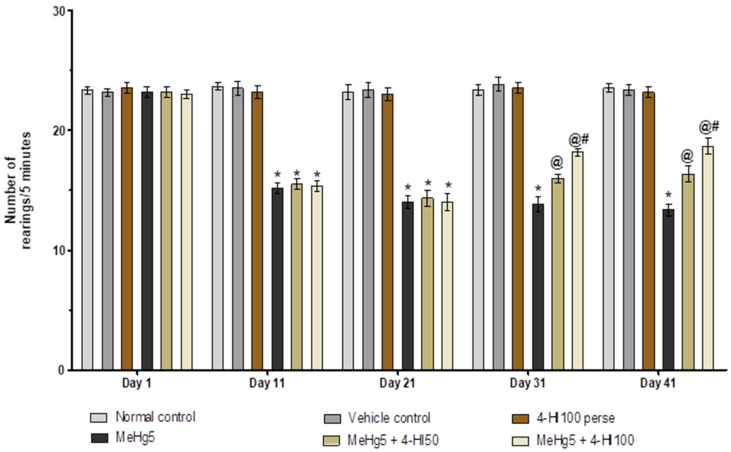
Effect of 4-hydroxyisoleucine on anxiety-like behaviour (number of rearing) after methyl mercuryexposure in rats.Statistical analysis by two-way ANOVA (posthoc Bonferroni’s test). Values are expressed as mean ± SEM (*n* = 6 rats per group). * *p* < 0.001 v/s normal control, vehicle control and 4-HI100 *perse*; @ *p* < 0.001 v/s MeHg5; @# *p* < 0.001 v/s MeHg5 + 4-HI50.

In order to evaluate general locomotor activity and examine the level of anxiety in rats, assessments of the number of boxes crossed and the number of rearings were performed on days 1, 11, 21, 31 and 41 of the protocol duration. On the first day, there was no significant difference between the groups. The MeHg-treated rats showed a progressive decline in the number of boxes crossed on days 11, 21, 31 and 41, and a considerable decrease in the number of rearings compared with normal control, vehicle control, and 4-HI100 *perse* groups. Consequently, the regular administration of 4-HI 50 mg/kg and 100 mg/kg significantly enhanced the locomotor activity (two-way ANOVA: F(20,120) = 35.71, *p* < 0.001) and restored the level of anxiety (rearing score)(two-way ANOVA: F(20,120) = 16.16, *p* < 0.001) as compared to MeHg-treated rats. On days 31 and 41, it was observed that 4-HI100 mg/kg considerably enhanced locomotion and improved the rearing scores when compared to the 4-HI 50 mg/kg treatment group (Figure 5 and Figure 6).

#### 3.2.3. Decreased Depression-Like Behaviour after Long-Term Administration of 4-Hydroxyisoleucine

**Figure 7:**
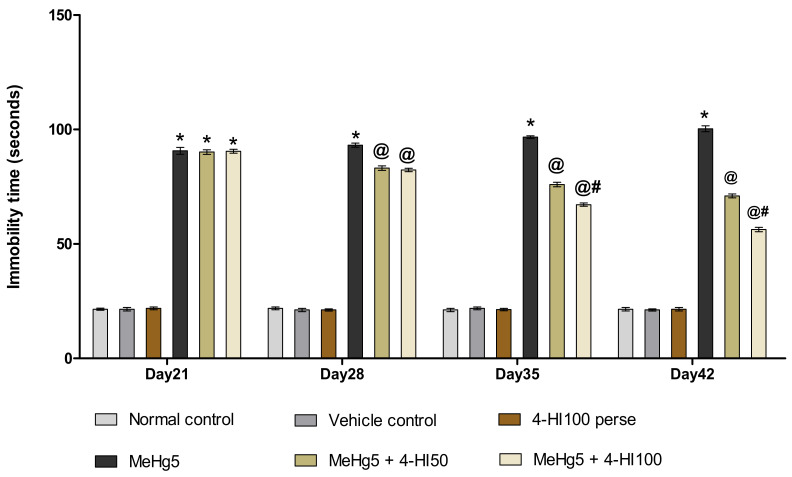
Effect of 4-hydroxyisoleucine on immobility time after methyl mercuryexposure in rats. Statistical analysis by two-way ANOVA (posthoc Bonferroni’s test). Values are expressed as mean ± SEM (*n* = 6 rats per group). * *p* < 0.001 v/s normal control, vehicle control and 4-HI100 *perse*; @ *p* < 0.001 v/s MeHg5; @# *p* < 0.001 v/s MeHg5 + 4-HI50.

The forced swim test (FST) was performed on days 21, 28, 35 and 42 of the protocol sequence to explore the despair behaviour in rats. The MeHg-treated group showed depressive-like behaviour and a gradual increase in immobility time compared to normal control, vehicle control, and 4-HI100 *perse* treated rats. Long-term oral administration of 4-HI50 mg/kg and 4-HI100 mg/kg led to a considerable decline in the immobility time on days 28, 35 and 42 when compared to the MeHg-treated group (two-way ANOVA: F(15,90) = 87.32, *p* < 0.001). Among these, 4-HI at a dose of 100 mg/kg was found to be more effective than 4-HI 50 mg/kg in reducing immobility time in rats on days 28, 35 and 42 (Figure 7).

#### 3.2.4. Improved Memory and Cognition after Long-Term Administration of 4-Hydroxyisoleucine

**Figure 8:**
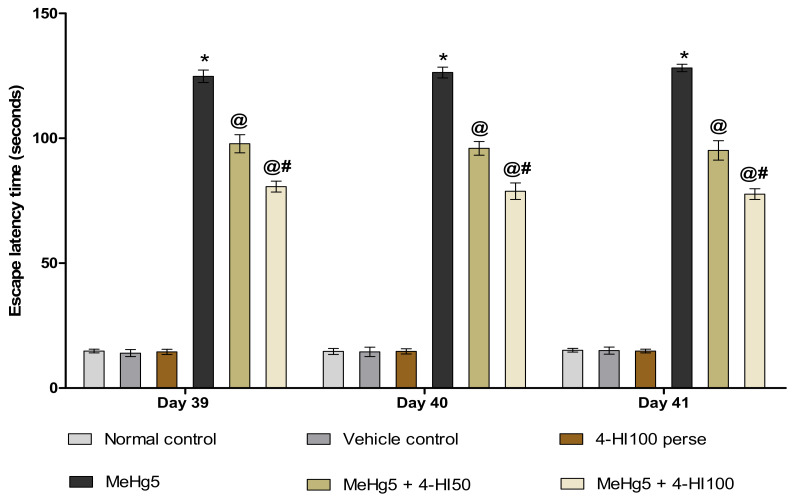
Effect of 4-hydroxyisoleucine on escape latency time (ELT) after methylmercuryexposure in rats.Statistical analysis by two-way ANOVA (posthoc Bonferroni’s test). Values are expressed as mean ± SEM (*n* = 6 rats per group). * *p* < 0.001 v/s normal control, vehicle control and 4-HI100 *perse*; @ *p* < 0.001 v/s MeHg5; @# *p* < 0.001 v/s MeHg5 + 4-HI50.

Escape latency time (ELT) was evaluated on the treatment schedule’s 39th, 40th and 41st days. MeHg-treated rats showed a gradual increase in ELT compared to the normal control, vehicle control, and 4-HI100 *perse* groups. Chronic treatment with 4-HI 50 mg/kg and 100 mg/kg resulted in a significant decrease in ELT compared to the MeHg-treated group (two-way ANOVA: F(10,60) = 1.891, *p* < 0.001). Administration of 4-HI100 mg/kg led to a marked decline in ELT compared to the 4-HI 50 mg/kg treatment, suggesting that 4-HI at a dose of 100 mg/kg is more efficient in improving memory acquisition in rats (Figure 8).

**Figure 9:**
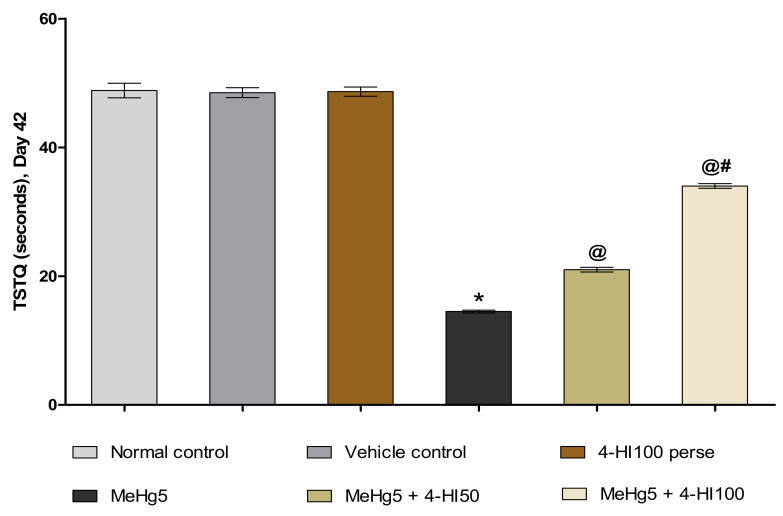
Effect of 4-hydroxyisoleucine on TSTQ after methylmercuryexposure in rats. Statistical analysis by one-way ANOVA (posthoc Tukey’s test). Values are expressed as mean ±SEM (*n* = 6 rats per group). * *p* < 0.001 v/s normal control, vehicle control and 4-HI100 *perse*; @ *p* < 0.001 v/s MeHg5; @# *p* < 0.001 v/s MeHg5 + 4-HI50.

TSTQ was also examined at the end of the treatment schedule (on day 42) to determine the degree of memory consolidation or memory retention in rats following learning. MeHg-induced rats demonstrated a more substantial decrease in time spent in the target zone than in the normal control, vehicle control, and 4-HI100 *perse* groups. Long-term oral treatment with 4-HI 50 mg/kg and 100 mg/kg comparatively increased the TSTQ compared to theMeHg-treated group (one-way ANOVA: F(5,25) = 1.967, *p* < 0.001). Relatively, 4-HI100 mg/kg showed a significant rise in TSTQ compared to 4-HI-50 mg/kg-treated rats (Figure 9).

### 3.3. Effect of 4-Hydroxyisoleucine on Neurochemical Alterations after Methyl MercuryExposure in Rats

#### 3.3.1. Increased Level of IGF-1 after Long-Term Administration of 4-Hydroxyisoleucine

**Figure 10:**
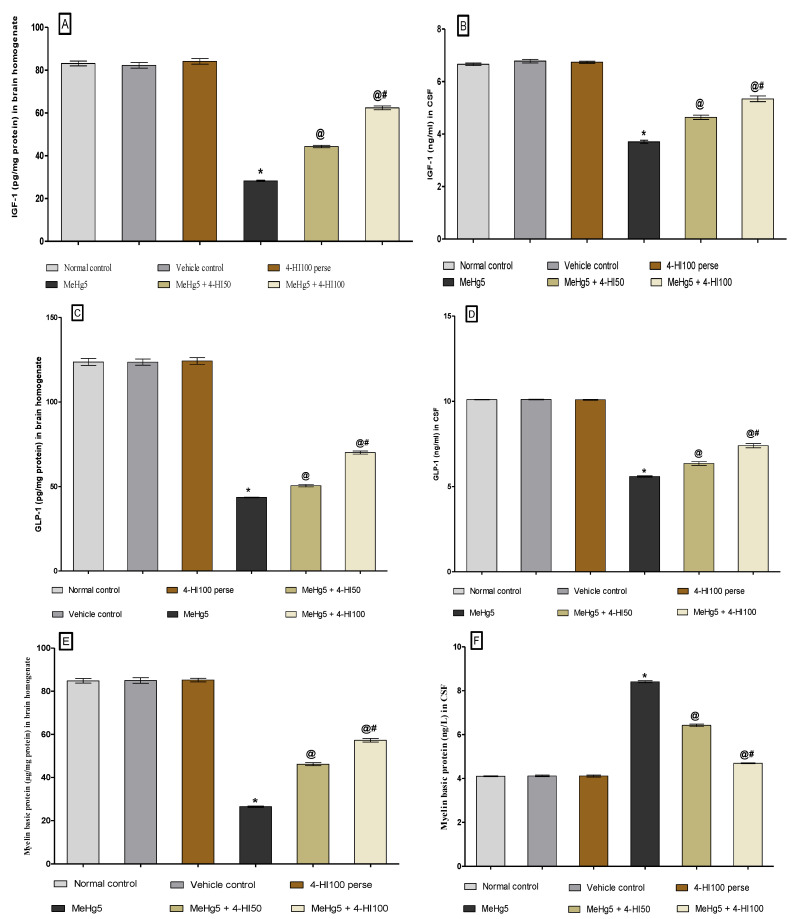
Effect of 4-hydroxyisoleucine on IGF-1, GLP-1 and myelin basic protein levels after methyl mercuryexposure in rats (**A**–**F**). Statistical analysis by one-way ANOVA (posthoc Tukey’s test). Values are expressed as mean ± SEM (*n* = 6 rats per group). * *p* < 0.001 v/s normal control, vehicle control and 4-HI100 *perse*; @ *p* < 0.001 v/s MeHg5; @# *p* < 0.001 v/s MeHg5 + 4-HI50.

IGF-1 levels were assessed in rat brain homogenate and CSF at the end of the experimental protocol. Oral administration of MeHg resulted in a significant decrease in the level of IGF-1 compared to the normal control, vehicle control, and 4-HI100 *perse* groups. Chronic oral administration of 4-HI at the doses of 50 mg/kg and 100 mg/kg remarkably increased the IGF-1 level in brain homogenate (one-way ANOVA: F(5, 25) = 5.595, *p* < 0.001) and CSF samples (one-way ANOVA: F(5,25) = 0.971, *p* < 0.001). Moreover, 4-HI100 mg/kg was more efficient than 4-HI50 mg/kg in restoring the IGF-1 protein level in rat brain homogenate and CSF samples (Figure 10A,B).

#### 3.3.2. Increased Level of GLP-1 after Long-Term Administration of 4-Hydroxyisoleucine

GLP-1 levels in rat brain homogenate and CSF were measured at the end of the treatment schedule to explore whether 4-HI activates GLP-1 signalling. MeHg-induced rats illustrated a remarkable decrease in the level of GLP-1 as compared to the normal control, vehicle control, and 4-HI100 *perse* treated groups. Prolonged 4-HI50 mg/kg and 100 mg/kg treatment led to a significant and dose-dependent increase in the GLP-1 concentration in brain homogenate (one-way ANOVA: F (5,25) = 2.009, *p* < 0.001) and CSF (one-way ANOVA: F(5,25) = 2.021, *p* < 0.001), respectively. Moreover, the 4-HI100 mg/kg dose was more efficient in restoring the level of GLP-1 in comparison with 4-HI50 mg/kg (Figure 10C,D).

#### 3.3.3. Restored Level of Myelin Basic Protein after Long-Term Administration of 4-Hydroxysoleucine

At the end of the protocol schedule, the level of myelin basic protein (MBP) was assessed in rat brain homogenate and CSF using a commercial ELISA kit. MBP levels were considerably lower in brain homogenate and higher in CSF samples in theMeHg-toxin-treated compared to the normal control, vehicle control, and 4-HI100 *perse* groups. In comparison to the MeHg-treated group, long-term treatment with 4-HI at doses of 50 mg/kg and 100 mg/kg enhanced MBP level in rat brain homogenate (one-way ANOVA: F(5,25) = 0.7205, *p* < 0.001) but lowered the MBP level in CSF (one-way ANOVA: F(5,25) = 0.5460, *p* < 0.001). It was also found that 4-HI100 mg/kg administration effectively restored the altered MBP levels compared to 4-HI50 mg/kg administration, implying that 4-HI is beneficial in restoring myelin degradation and lowering demyelination in a dose-dependent manner (Figure 10E,F).

#### 3.3.4. Decreased Levels of Caspase-3 and Bax as well asIncreased Bcl-2 Levels after Long-Term Administration of 4-Hydroxyisoleucine

**Figure 11:**
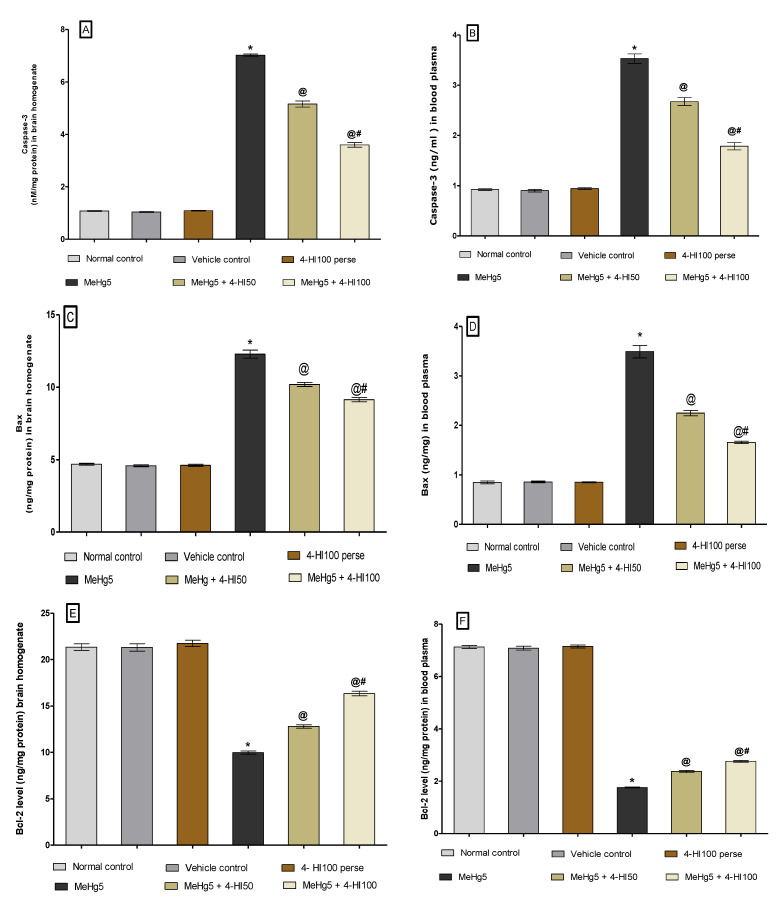
Effect of 4-hydroxyisoleucine on caspase-3, Bax and Bcl-2 levels after methyl mercuryexposure in rats (**A**–**F**). Statistical analysis by one-way ANOVA (posthoc Tukey’s test). Values are expressed as mean ± SEM (*n* = 6 rats per group). * *p* < 0.001 v/s normal control, vehicle control and 4-HI100 *perse*; @ *p* < 0.001 v/s MeHg5; @# *p* < 0.001 v/s MeHg5 + 4-HI50.

At the end of the protocol schedule, the neuronal cell death markers such as caspase-3, Bax, and Bcl-2 were evaluated in rat brain homogenate and blood plasma. Long-term oral MeHg administration significantly increased pro-apoptotic markers like caspase-3 and Bax in rat brain homogenate and blood plasma. In contrast, oral treatment with MeHg for 21 days led to a notable decline in anti-apoptotic Bcl-2 protein level in rat brain homogenate and blood plasma compared to normal control, vehicle control, and 4-HI-*perse*-treated groups. Chronic oral administration of 4-HI at doses of 50 mg/kg and 100 mg/kg substantially reduced the level of caspase-3 in brain homogenate (one-way ANOVA: F(5,25) = 0.9588, *p* < 0.001) and blood plasma (one-way ANOVA: F(5,25) = 0.5116, *p* < 0.001),respectively (Figure 11A,B).

Likewise, continuous oral treatment with 4-HI at doses of 50 mg/kg and 100 mg/kg remarkably decreased the level of the pro-apoptotic marker Bax in rat brain homogenate (one-way ANOVA: F(5,25) = 3.902, *p* < 0.001)and blood plasma (one-way ANOVA: F(5,25) = 1.642, *p* < 0.001) (Figure 11C,D).

However, regular oral administration of 4-HI at doses of 50 mg/kg and 100 mg/kg for 21 days consecutively led to a considerable increase in the level of Bcl-2 protein in brain homogenate (one-way ANOVA: F(5,25) = 7.161, *p* < 0.001) and blood plasma (one-way ANOVA: F(5,25) = 0.9438, *p* < 0.001) in the MeHg-treated rats. Furthermore,4-HI100 mg/kg treatment was more effective than 4-HI50 mg/kg treatment in restoring the abnormal levels of apoptotic markers in rats (Figure 11E,F).

#### 3.3.5. Restoration of Neurotransmitter Levels after Long-Term Administration of 4-Hydroxyisoleucine

The amounts of neurotransmitters such as GABA, glutamate, acetylcholine, and serotonin in the rat brain homogenate were estimated at the end of the protocol schedule. Oral administration of the toxin MeHg for 21 days consecutively resulted in a substantial decrease in GABA, acetylcholine, and serotonin levels. In contrast, continuous oral MeHg intoxication led to a significant increase in the glutamate concentration in brain homogenate compared with normal control, vehicle control, and 4-HI100-*perse*-treated rats.

Treatment with 4-HI at doses of 50 mg/kg and 100 mg/kg considerably and dose-dependently raised the levels of GABA (one-way ANOVA: F(5,25) = 3.337, *p* < 0.001), acetylcholine (one-way ANOVA: F(5,25) = 0.3535, *p* < 0.001), and serotonin (one-way ANOVA: F(5,25) = 1.245, *p* < 0.001) in the brain homogenate of MeHg-treated rats.

**Figure 12:**
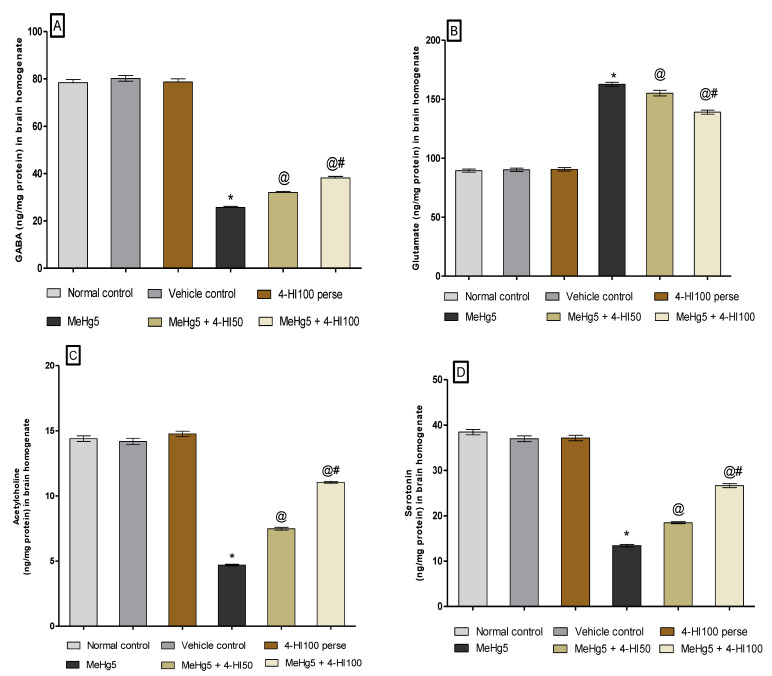
Effect of 4-hydroxyisoleucine on neurotransmitters after methyl mercuryexposure in rats (**A**–**D**). Statistical analysis by one-way ANOVA (posthoc Tukey’s test). Values are expressed as mean ± SEM (*n* = 6 rats per group). * *p* < 0.001 v/s normal control, vehicle control and 4-HI100 *perse*; @ *p* < 0.001 v/s MeHg5; @# *p* < 0.001 v/s MeHg5 + 4-HI50.

However, oral administration of 4-HI at doses of 50 mg/kg and 100 mg/kg lowered the concentration of glutamate (one-way ANOVA: F(5,25) = 1.372, *p* < 0.001) in rat brain homogenate in contrast to the MeHg-treated group. Among these, the most significant improvements were observed in 4-HI100-mg/kg-treated rats versus the 4-HI50-mg/kg-treated rats in restoring neurotransmitter imbalance (Figure 12A–D).

#### 3.3.6. Reduction in Neuroinflammatory Cytokines after Long-Term Administration of 4-Hydroxyisoleucine

**Figure 13:**
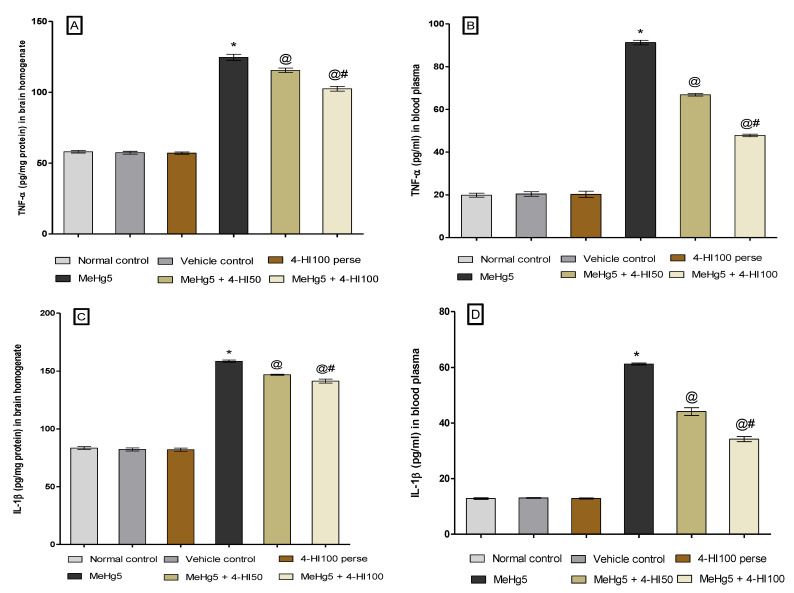
Effect of 4-hydroxyisoleucine on neuroinflammatory cytokines after methyl mercuryexposure in rats (**A**–**D**). Statistical analysis by one-way ANOVA (posthoc Tukey’s test). Values are expressed as mean ± SEM (*n* = 6 rats per group). * *p* < 0.001 v/s normal control, vehicle control and 4-HI100 *perse*; @ *p* < 0.001 v/s MeHg5; @# *p* < 0.001 v/s MeHg5 + 4-HI50.

To examine the neuroprotective effect of 4-HI on the pro-inflammatory cytokines, we evaluated the level of TNF-α and IL-1β in rats’ whole brain homogenate and blood plasma. Treatment with 4-HI at doses of 50 mg/kg and 100 mg/kg significantly lessened the TNF-α level in rat brain homogenate (one-way ANOVA: F(5,25) = 3.181, *p* < 0.001) and blood plasma (one-way ANOVA: F(5,25) = 1.682, *p* < 0.001), respectively. Similarly, chronic oral treatment with 4-HI50 mg/kg and 4-HI100 mg/kg remarkably lowered the level of IL-1β in brain homogenate (one-way ANOVA: F(5,25) = 2.058, *p* < 0.001) and blood plasma (one-way ANOVA: F(5,25) = 1.370, *p* < 0.001) as opposed to the MeHg-toxin-administered rats. Meanwhile, 4-HI100 mg/kg treatment showed a marked improvement in reducing the level of these neuroinflammatory cytokines compared to the 4-HI50 mg/kg dose (Figure 13A–D).

#### 3.3.7. Restored Antioxidant Levels after Long-Term Administration of 4-Hydroxyisoleucine

At the end of the experimental protocol, oxidative stress markers like AchE, LDH, MDA, nitrite, SOD, and GSH were quantified in rat brain homogenate. Long-term treatment withMeHg at a dose of 5 mg/kg for 21 dayson rats resulted in a considerable increase in AchE, LDH, MDA, and nitrite concentrations. In contrast, oral MeHg administration led to a substantial decline in antioxidants like SOD and GSH compared to normal control, vehicle control, and 4-HI100-*perse*-treated groups. Chronic treatment of three weeks with 4-HI at doses of 50 mg/kg and 100 mg/kg significantly lowered the levels of AchE(one-way ANOVA: F(5,25) = 2.460, *p* < 0.001), LDH (one-way ANOVA: F(5,25) = 4.095, *p* < 0.001), MDA (one-way ANOVA: F(5,25) = 1.081, *p* < 0.001), and nitrite (one-way ANOVA: F(5,25) = 2.286, *p* < 0.001). 

**Figure 14:**
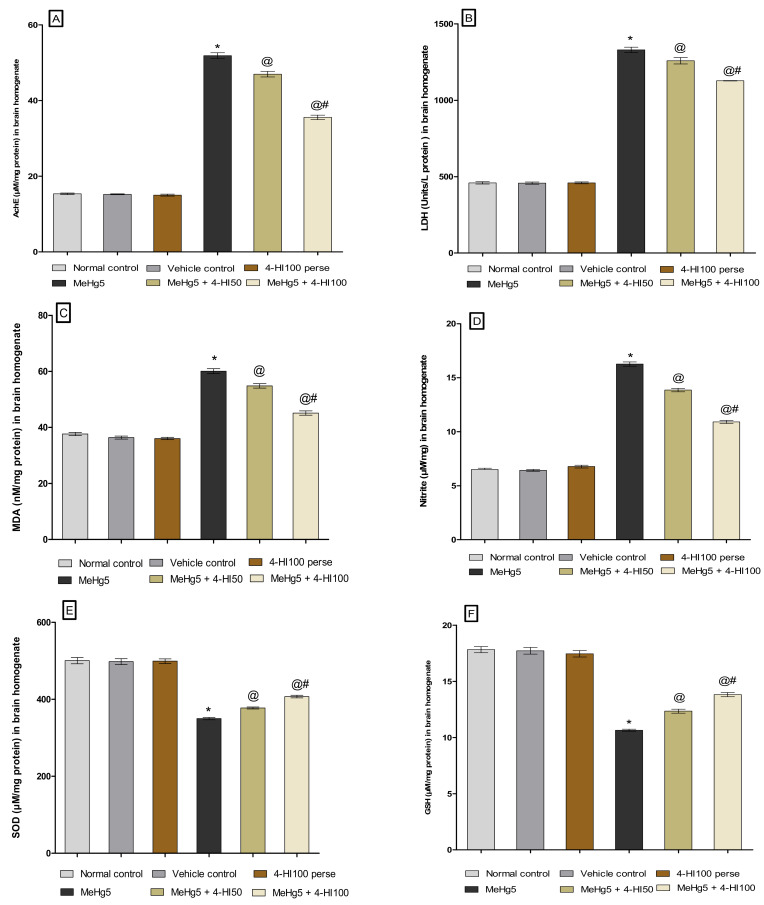
Effect of 4-hydroxyisoleucine on oxidative stress markers after methyl mercuryexposure in rats (**A**–**F**). Statistical analysis by one-way ANOVA (posthoc Tukey’s test). Values are expressed as mean ± SEM (*n* = 6 rats per group). * *p* < 0.001 v/s normal control, vehicle control and 4-HI100 *perse*; @ *p* < 0.001 v/s MeHg5; @# *p* < 0.001 v/s MeHg5 + 4-HI50.

However, 4-HI50 mg/kg and 100 mg/kg remarkably restored the anti-oxidant defence system by considerably increasing the levels of reduced glutathione (one-way ANOVA: F(5,25) = 1.226, *p* < 0.001) and SOD (one-way ANOVA: F(5,25) = 0.8858, *p* < 0.001) when compared with MeHg-treated rats. Compared to 4-HI50 mg/kg, the 4-HI100 mg/kg treatment significantly reduced oxidative stress markers while restoring antioxidant levels in a dose-dependent manner (Figure 14A–F).

### 3.4. Effect of 4-HI on Gross Pathological Alterations and Demyelination Volume after Methyl MercuryExposure in Rats

#### 3.4.1. Improvement in Whole-Brain Morphological Alterations after Long-Term Administration of 4-Hydroxyisoleucine

**Figure 15:**
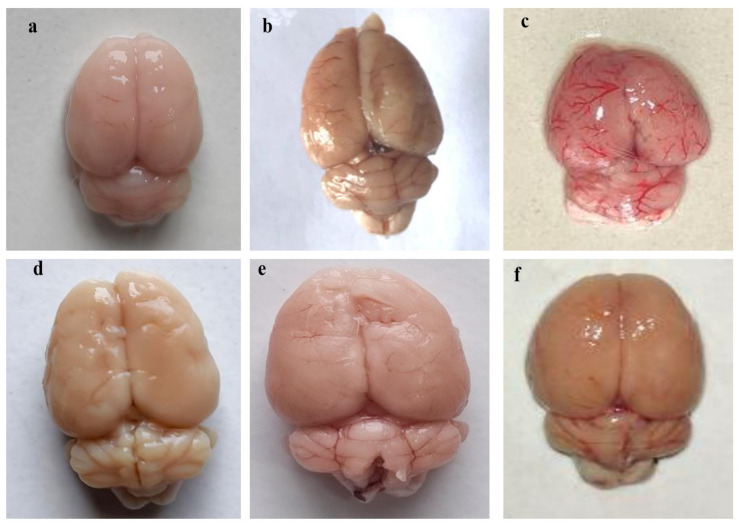
The effect of 4-hydroxyisoleucine on whole brain morphology in rats following methyl mercury exposure. (**a**) Normal control, (**b**) Vehicle control, (**c**) 4-HI100 *perse*, (**d**) MeHg5, (**e**) MeHg5 + 4-HI50, (**f**) MeHg5 + 4-HI100.

The normal control, vehicle control, and 4-HI-*perse*-treated groups showed appropriate brain morphology, adequate shape, and unaltered size. MeHg-treated rat brains showed shrinkage in size and reduced overall brain mass compared to normal control, vehicle control, and 4-HI-*perse*-treated groups. Continuous oral administration of 4-HI at doses of 50 mg/kg and 100 mg/kg was observed to be efficient in restoring the alterations in brain weight and effectively reducing cortical degeneration. However, 4-HI100-mg/kg-treated rats showed significant improvements in whole-brain morphology compared to the 4-HI50 mg/kg treated rats (Figure 15).

#### 3.4.2. Reduced Pathological Abnormalities in Brain Sections after Long-Term Administration of 4-Hydroxyisoleucine

**Figure 16:**
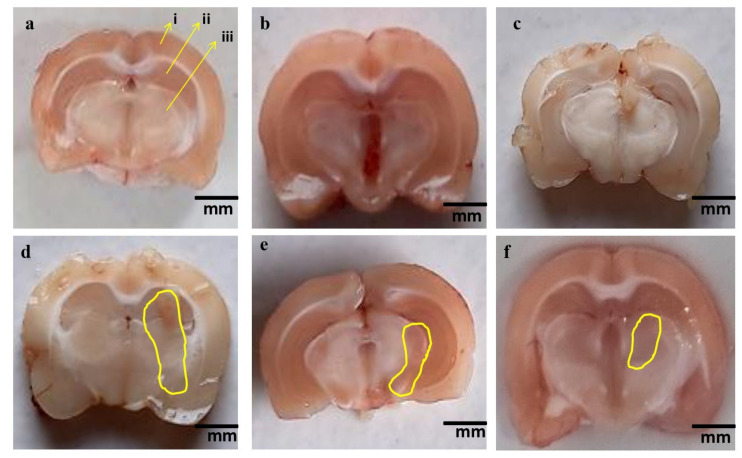
The effect of 4-hydroxyisoleucine on gross pathological changes (brain sections) in rats following methyl mercury exposure. (**a**) Normal control [i. Cerebral cortex, ii. Hippocampus, iii. Basal ganglia] (**b**) Vehicle control. (**c**) 4-HI100 *perse*. (**d**) MeHg5. (**e**) MeHg5 + 4-HI50. (**f**) MeHg5 + 4-HI100. *Note: Yellow circles point to the demyelinated area; (Scale bar:5 mm)*.

The normal control, vehicle control, and 4-HI100-*perse*-treated rat brain sections had distinct appearances, with clearly defined basal ganglia, cerebral cortex, and hippocampal tissues. However, the brain sections of rats treated with MeHg demonstrated a considerable reduction in several cortical tissues, myelin degradation, white matter degeneration, and demyelination compared to normal control, vehicle control, and 4-HI100-*perse*-treated rats. Persistent treatment with 4-HI50 mg/kg and 4-HI100 mg/kg remarkably reversed these morphological alterations in brain sections and restored demyelination, measured by demyelination volume and MBP level assessment. Among these, 4-HI100 mg/kg was more effective than 4-HI50 mg/kg in ameliorating the pathological abnormalities in brain sections (Figure 16).

#### 3.4.3. Reduced Demyelination Volume after Long-Term Administration of 4-Hydroxyisoleucine

**Figure 17:**
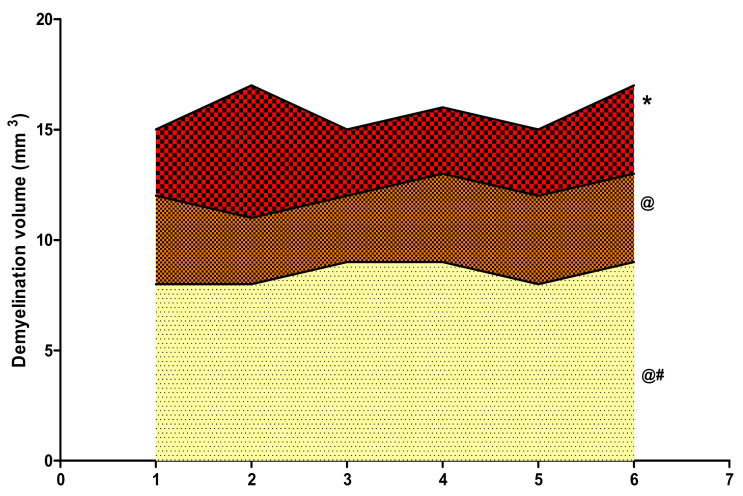
Effect of 4-hydroxyisoleucine on demyelination volume after methyl mercuryexposure in rats. Statistical analysis by one-way ANOVA (posthoc Tukey’s test). Values are expressed as mean ± SEM (*n* = 6 rats per group). * *p* < 0.001 v/s normal control, vehicle control and 4-HI100 *perse*; @ *p* < 0.001 v/s MeHg5; @# *p* < 0.001 v/s MeHg5 + 4-HI50.

Long-term oral administration of the neurotoxin MeHg for 21 days substantially induced the destruction of the myelin content compared to normal control, vehicle control, and 4-HI100-*perse*-treated animals. Treatment with 4-HI at doses of 50 mg/kg and 100 mg/kg considerably reduced the demyelination volume relative to MeHg-treated rats (one-way ANOVA: F(5,25) = 1.627, *p* < 0.001). Consequently, 4-HI100 mg/kg showed a noticeable, dose-dependent improvement in myelin restoration compared to 4-HI50 mg/kg, as further evaluated by demyelination volume (Figure 17).

#### 3.4.4. Effect of 4-HI onMethyl-Mercury-Induced Histopathological Changes

**Figure 18:**
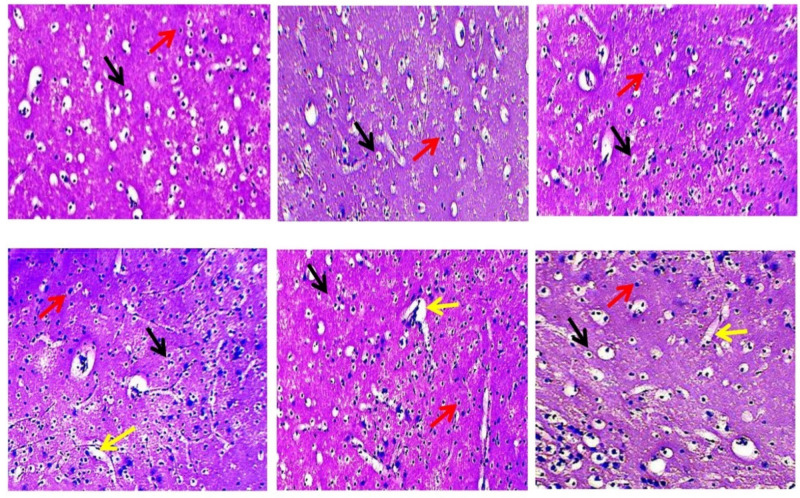
The effect of 4-hydroxyisoleucine on histopathological alterations in rats following methyl mercury exposure. The slides were selected to show neuronal populations stained with hematoxylin and eosin under a fluorescence microscope. Light microphotographs of the cerebral cortex revealed the following morphological changes during MeHg exposure and 4-HI treatment: ‘Section: A’ shows the vehicle group.The black arrows indicate healthy oligodendrocyte cells, mostly found in the cerebral cortex. The red arrow indicates microglia. ‘Section: B’ shows the sham control group, with black arrows indicating healthy oligodendrocyte cells and red arrows signifying microglia. ‘Section: C’ shows the 4-HI-perse-treated group, which exhibits the typical pattern of all neuronal cells, just like the sham control group. ‘Section: D’ depicts a MeHg-treated group with oligodendrocyte degeneration and necrosis, as indicated by the black and yellow arrows, as well as an increase in microglial density (red arrow). ‘Section: E’ represents theMeHg- and 4-HI-(50 mg/kg)-treated group showing regeneration of oligodendrocytes represented by the black arrow, decreased density of microglial cells represented by the red arrow, and recovery of neuronal cells by decreasing the area of necrosis represented by the yellow arrow. ‘Section: F’ depicts the diminished area of necrosis indicated by the yellow arrow in the MeHg- and 4-HI-(100 mg/kg)-treated group, the black arrow represents the restoration of oligodendrocyte, and the red arrow represents reduced microglial density.

The vehicle control, sham control, and 4-HI*-perse*-treated groups showed normal morphology of the cerebral cortex neurons characterised by rod-shaped microglial cells and healthy oligodendrocytes with no area of necrosis. The methyl-mercury-treated group showed destruction of oligodendrocytes cells and the activation of microglia, and an increase in the area of necrosis. With4-HI at a dose of 50 mg/kg, a gradual repair in the oligodendrocytes cells along with a slight decrease in the number of astrocytes was visible. The dose of4-HI 100 mg/kg restored the shape of oligodendrocyte cells and decreased the microglial and necrosis areas (Figure 18).

## 4. Discussion

The current study investigated pathological alterations similar to ALS using an experimental rat model of MeHg+ poisoning. Several animal studies have suggested that MeHg+ exposure may contribute to ALS progression [1,4,82]. In a mouse model with mutant human SOD1 gene overexpression, MeHg+ exposure elicited ALS-like symptoms, including the early development of hind limb paralysis [83].

With its lipophilicity, MeHg+ can quickly diffuse into the central nervous system from the bloodstream [84]. It forms covalent bonds with the sulfhydryl (thiol) groups of plasma cholinesterase [85]. Various neurobehavioral tests were used to determine whether 4-HI has neuroprotective effects in MeHg+-treated rats. We also investigated cellular and molecular markers, apoptotic markers, neurotransmitters, pro-inflammatory cytokine levels, and oxidative stress indicators in rat brain homogenate, blood plasma, and CSF.

In rats treated with MeHg+, oral administration of 4-HI at doses of 50 mg/kg and 100 mg/kg showed a substantial neuroprotective effect against motor dysfunctions and neurological impairments. Chronic exposure to 5 mg/kg MeHg+ resulted in a significant decrease in animal body weight and the relative brain-to-body weight ratio. After prolonged oral treatment with 4-HI, however, bodyweight was gradually restored. The open-field task (OFT) assessed rats’ general locomotor activity and exploratory behaviour changes [53]. MeHg exposure resulted in a significant reduction in the number of boxes crossed. Furthermore, 4-HI therapy remarkably alleviated locomotive impairment and anxiety-like symptoms in ALS-like rats.

Our findings indicate that repeated 4-HI treatment eventually eliminates depressive-like states in MeHg-exposed rats, suggesting that it has promising neuroprotective effects in restoring brain function. MeHg has a negative effect on neuronal plasticity, learning, and memory, according to research [86,87]. Our study examined the effects of 4-HI on long-term memory and cognitive behaviour. Long-term drug administration reversed these neuropathologies in rats, indicating significant improvements in memory acquisition and retention.

The IGF-1/GLP-1 signalling pathway controls neuronal activity, excitotoxicity [26], neuroinflammation, oxidative stress [88], oligodendrogenesis [89], and myelin sheath synthesis [90]. This pathway disruption is detrimental to the progression of ALS pathogenesis and related neuro-complications [91]. The levels of cellular and molecular markers such as IGF-1 and GLP-1 in rat brain homogenate and CSF were determined using ELISA. Oral 4-HI therapy effectively restored IGF-1 and GLP-1 protein levels in rat brain homogenate and CSF. These findings imply that 4-HI can modulate the dysregulation of IGF-1/GLP-1 signalling in damaged neuronal cells [48].

Oligodendrocytes are glial cells that create myelin sheaths, which act as electrical insulators and aid in maintaining axonal homeostasis [92]. Demyelination has been associated with motor dysfunction, neurological impairments, lower cognitive abilities, and decreased electrical impulse conduction velocity in neurons [93].

It has been discovered that white matter damage and demyelination occur before motor neuron death and can be used to predict ALS prognosis [94]. A study examined oligodendrocyte degeneration in the spinal cords of G93A-SOD1 ALS mice before the progression of the disease. Owing to oligodendrocyte dysfunction, even though new oligodendrocytes were produced, they failed to mature, increasing demyelination [95]. Another study discovered decreased MBP levels in the spinal cords of ALS patients after death [96]. The oral administration of MeHg over a long period resulted in significant changes in MBP content in brain homogenates and CSF of rats. A 4-HI treatment resulted in the gradual, dose-dependent restoration of impaired MBP levels in the individual samples. Multiple neuropathogenic mechanisms have been identified as altering neuronal homeostasis and activate cell apoptotic cascades, resulting in motor neuron death [97]. Apoptosis-related indicators such as caspase-3 and Bax gradually increased in rat brains following oral MeHg administration. In contrast, after long-term treatment with 4-HI, the levels of these apoptotic markers in rat brain homogenate and blood plasma were seen to be reversed in a dose-dependent manner, in both the brain and the blood plasma samples. These findings suggest that 4-HI may be capable of slowing the progression of ALS by preventing the loss of motor neurons.

Glutamate excitotoxicity has been associated with the neurodegenerative presentation of ALS [98,99], the death of oligodendrocytes, and the degeneration of white matter in the spinal cord [100]. Increased glutamate (an excitatory mediator) levels have been reported in the motor cortex and white matter of ALS patients [101]. Exposure to MeHg significantly decreased GABA, acetylcholine, and serotonin levels while significantly increasing glutamate levels, suggesting glutamate-mediated excitotoxic effects. The dose-dependent administration of 4-HI significantly restored the altered concentrations of these neurotransmitters in the brain. Neuronal inflammation has been identified as a significant pathogenic feature of ALS. Cytokine-induced inflammation may play an essential role in the pathophysiology of ALS [102,103]. TNF-α, IL-1β, and IL-6are among the inflammatory cytokines increased by MeHg exposure [104]. The presence of TNF-α and IL-1β in MeHg-treated rats enhanced the levels of these inflammatory mediators. Chronic oral treatment with 4-HI, on the other hand, reduced the altered inflammatory cytokine levels in rat brain homogenate and blood plasma samples.

MeHg has been shown to promote the formation of reactive oxygen species (ROS) in the brain, leading to increased ROS synthesis [104]. This is consistent with prior research indicating the essential role of oxidative stress in the pathogenesis of ALS [105,106]. The development of an ALS-like phenotype in rats resulted in increased oxidative stress markers such as LDH, MDA, and nitrite. Long-term 4-HI therapy significantly reduced the amounts of these oxidative metabolites. This shows that 4-HI may act as an antioxidant, lowering the risk of oxidative damage.

Numerous studies have linked the loss of myelin in the white matter tracts and oligodendroglial involvement to the onset of ALS [107]. A morphometric study was conducted to determine volumetric variations in the whole brain. It was found that ALS patients have a lower whole-brain volume than healthy control individuals [108]. Related research reveals that patients with ALS had a significantly lower white matter volume than the control group [109]. A similar study found significant atrophy of the white matter in ALS subgroups [110]. Treatment with 4-Hydroxyisoleucine (4-HI) restored gross morphological deficits throughout the brain and significantly reduced pathological alterations in brain sections. Dose-dependently, 4-HI significantly improved myelin restoration and decreased demyelination volume.

Our findings indicate that 4-HI protects the cerebral cortex of rat brains from neurotoxicity induced by MeHg. The present results indicate that the density of neuroglial cells was restored when 4-HI was administered at doses of 50 mg/kg and 100 mg/kg. In contrast, histology revealed that structural integrity was lost in the MeHg-treated group. In addition, it was discovered that 4-HI therapy at doses of 50 mg/kg and 100 mg/kg was effective in restoring neuroglial cells. By altering IGF-1/GLP signalling, the primary objective of the 4-HI research was to ameliorate neurobehavioral and neurochemical deficits in MeHg-treated rats. According to the current findings, IGF-1, GLP-1, and MBP levels can be employed as reliable early diagnostic biomarkers to predict a significant degenerative component of ALS. Above all, a mechanistic approach must be validated using knock-in/knock-out investigations of the IGF-1 and GLP-1 genes. Despite these limitations, the potency of 4-HI’s neuroprotective effect may lead to the development of a disease-modifying therapeutic intervention for ALS.

### Limitations

The IGF-1/GLP-1 cascade is essential for cell proliferation, differentiation, adhesion, migration, and survival. Consequently, it is involved in a wide range of physiological functions. To discover the molecular basis for this strategy, qualitative analyses of cellular markers, such as Western blotting and immunohistopathology, would be necessary. Given these constraints, 4-hydroxyisoleucine’s ability to restore the IGF-1/GLP-1 signalling cascade in the brain may allow the development of a possible treatment strategic plan for this neurodegenerative disease. We could also assess the extent of demyelination in MeHg-induced rats by using LFB (Luxol Fast Blue) dye.

## 5. Conclusions

Our findings show that 4-HI interacts with IGF-1/GLP-1 signalling and influences pathological abnormalities. The dual activation of IGF-1 and GLP-1 signalling by 4-HI in MeHg+-treated rats has yet to be studied pre-clinically. Our findings suggest that 4-HI may be a promising therapeutic drug candidate for treating behavioural, biochemical, neurochemical, and gross morphological alterations associated with the ALS-like phenotype in MeHg+-treated adult Wistar rats. Based on our histology findings, we may conclude that MeHg+ is a neurotoxin harmful to neuroglial cells. In the cerebral cortical regions of the rat brain, MeHg+ caused ALS, which is characterised by the death of oligodendrocytes and an increase in the microglial density and necrosis area. A 4-HItherapy at 50 mg/kg and 100 mg/kg doses prevents the toxic effect of MeHg+ and restores the structural integrity of oligodendrocytes, microglial cells, and the area of necrosis. These novel findings may be proposed as potential diagnostic biomarkers for finding a disease-modifying therapeutic moiety. However, more genetic and immuno-histological research is needed to describe the underlying pathways.

## Data Availability

All data generated or analysed during this study are included in this article. There are no separate or additional files.

## Abbreviations

Ach	Acetylcholine
AchE	Acetylcholinesterase
AkT	Protein kinase B
ALS	Amyotrophic lateral sclerosis
ALT	Alanine transaminase
ANOVA	Analysis of variance
AST	Aspartate transaminase
Bax	Bcl-2 associated X protein
Bcl-2	B cell lymphoma-2
BDNF	Brain-derived growth factor
cAMP	Cyclic AMP
Caspase-3	Cysteine-aspartic proteases, cysteine aspartases or cysteine-dependent aspartate-directed proteases-3
CNS	Central nervous system
CSF	Cerebrospinal fluid
ELT	Escape latency time
FST	Forced swim test
GABA	Gamma amino butyric acid
GLP-1	Glucagon like peptide-1
GLP-1R	Glucagon like peptide-1 receptor
GLUT	Glucose transporter
GSH	Reduced glutathione
GST	Grip strength test
HDL	High-density lipoprotein
IBD	Intestinal bowel disease
IGF-1	Insulin-like growth factor-1
IGF-1R	Insulin-like growth factor-1 receptor
IL-1β	Interleukin-1β
IRS-1	Insulin receptor substrate-1
LDH	Lactate dehydrogenase
LDL	Low-density lipoprotein
LPS	Lipopolysaccharide
MBP	Myelin basic protein
MDA	Malondialdehyde
MeHg	Methylmercury
MND	Motor neuron disease
MS	Multiple sclerosis
MWM	Morris water maze
NGF	Nerve growth factor
NO_2_	Nitrite
ODC	Oligodendrocytes
PC12	Pheochromocytoma cell 12
PI3K	Phosphoinositol 3-kinase
rh-IGF-1	Recombinant human-IGF-1
ROS	Reactive oxygen species
SMA	Spontaneous motor activity
SOD-1	Superoxide dismutase 1
TACE	TNF-α converting enzyme
TG	Triglycerides
TNF-α	Tumor necrosis factor-α
TSTQ	Time spent in target quadrant
4-HI	4-hydroxyisoleucine
5-HIAA	5-hydroxy indole acetic acid
5-HT	Serotonin
